# Supplementary material for: Inducible Systemic Gcn1 Deletion in Mice Leads to Transient Body Weight Loss upon Tamoxifen Treatment Associated with Decrease of Fat and Liver Glycogen Storage
Source: Int J Mol Sci. 2022 Mar 16;23(6):3201. doi: 10.3390/ijms23063201 (PMC8949040; doi:10.3390/ijms23063201)
Supplement: Supplementary file 1 [file ijms-23-03201-s001.zip › ijms-1624234-supplementary.pdf]

International Journal of
Molecular Sciences

Supplemental figures

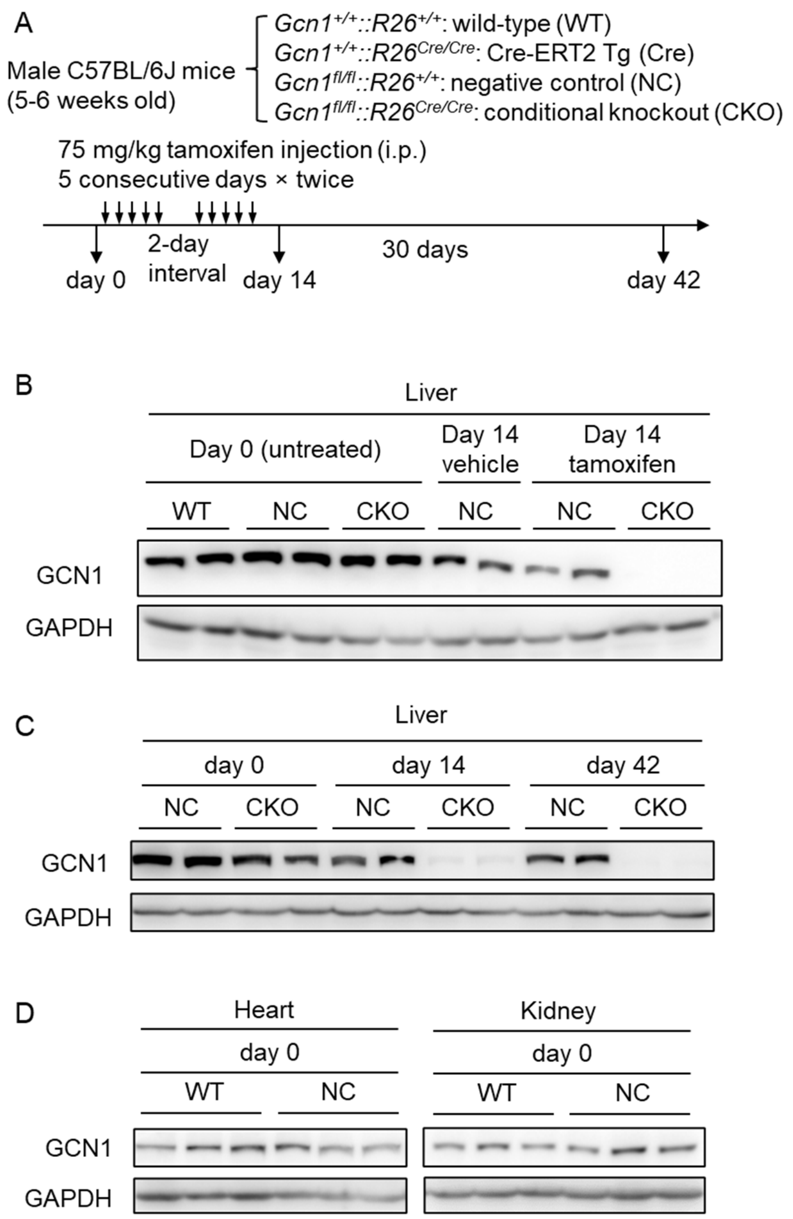

Figure S1. Tamoxifen injection protocol and GCN1 expression in liver. (**A**) Schematic regimen of tamoxifen injections. Tamoxifen (75 mg/kg) or oil (3.75 mL/kg) were injected into mice 10 times (five consecutive days with a 2-day interval). Mice were dissected at day 0 (without any treatment), day 14 (2 days after the last injection), and day 42 (30 days after the last injection). (**B**) Immunoblotting of Gcn1 expression in wild type (WT), negative control (NC), and conditional knockout (CKO) mice. (**C**) GCN1 expression in the liver of NC and CKO mice was compared at days 0, 14, and 42 after tamoxifen treatment. (**D**) GCN1 expression in the heart and kidney of NC and CKO mice was compared at day 0.

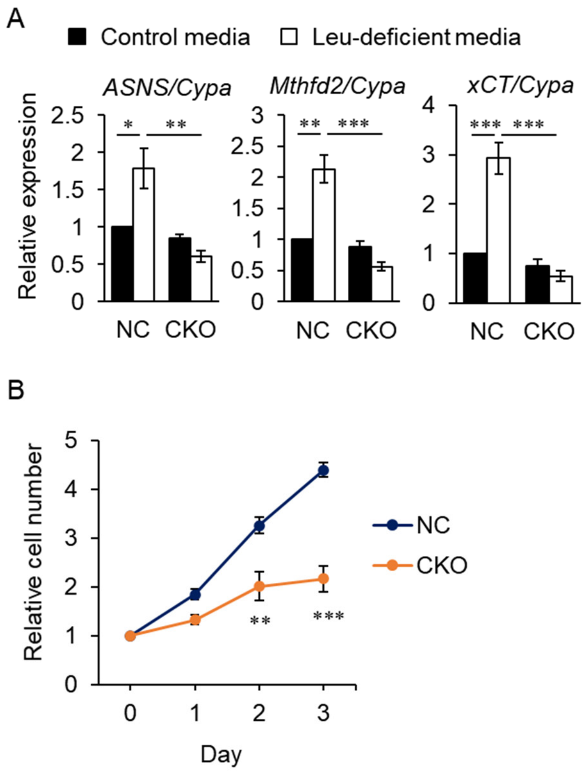

Figure S2. *Gcn1* CKO MEFs show impaired amino acid starvation response and decreased cell proliferation. (**A**) Amino acid starvation response of *Gcn1* CKO MEFs. Primary MEFs from *Gcn1*$^{fl/fl}$*::R26*$^{Cre/Cre}$ mice were treated with 4-hydoxytamoxifen (CKO) or vehicle (NC) for 8 days. Both MEFs were treated with Leu-deficient media or control media for 16 h. RT-qPCR was used to analyze the expression of the ATF4 target genes *ASNS*, *Mthfd2*, and *xCT*. The data were calculated by the ΔΔCt method and normalized to the internal control, *Cypa*. The value for NC MEFs treated with control media was set to 1. (**B**) Cell proliferation of NC and CKO MEFs. Relative cell number was assessed by Cell Counting Kit-8 and data are presented as mean ± SEM of three independent experiments. Statistical analysis was carried out using two-way ANOVA and Tukey's test. *$p < 0.05$, **$p < 0.01$, ***$p < 0.001$.

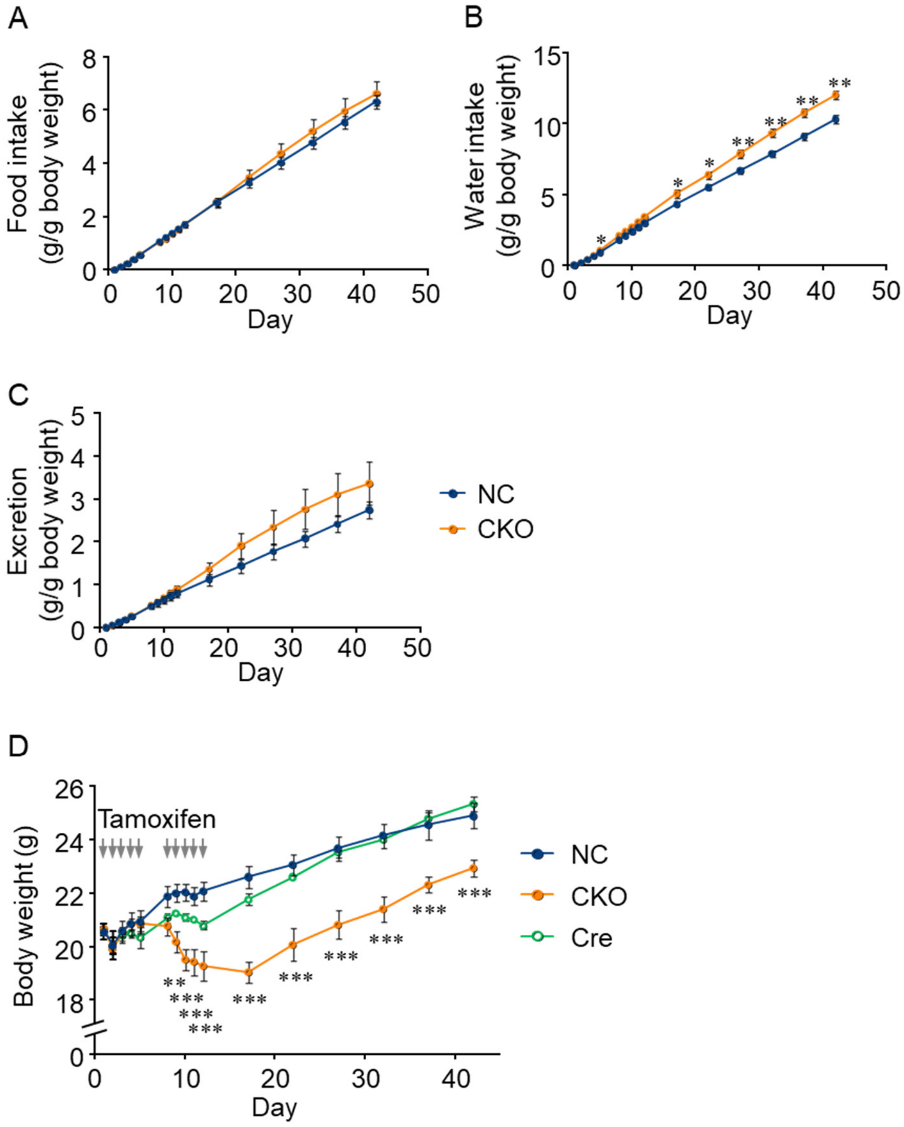

Figure S3. Changes in food/water intake and excretion, and body weight change in CreERT2 knock-in mice. (**A–C**) Changes in food intake (**A**), water intake (**B**), and excretion (**C**) of NC and CKO mice. Data were recorded during and after tamoxifen injections and normalized to the body weight. Data are expressed as mean ± SEM ($n = 6$). (**D**) Body weight change of CreERT2 knock-in (*Gcn1*$^{+/+}$::*R26*$^{Cre/Cre}$) mice upon tamoxifen injections ($n = 3$) was compared with the data of NC ($n = 6$) and CKO mice ($n = 6$) shown in Fig 3A. Data are expressed as mean ± SEM. Statistical significance was analyzed using two-way ANOVA and Tukey's test (*$p < 0.05$, **$p < 0.01$, ***$p < 0.001$ compared with NC group).

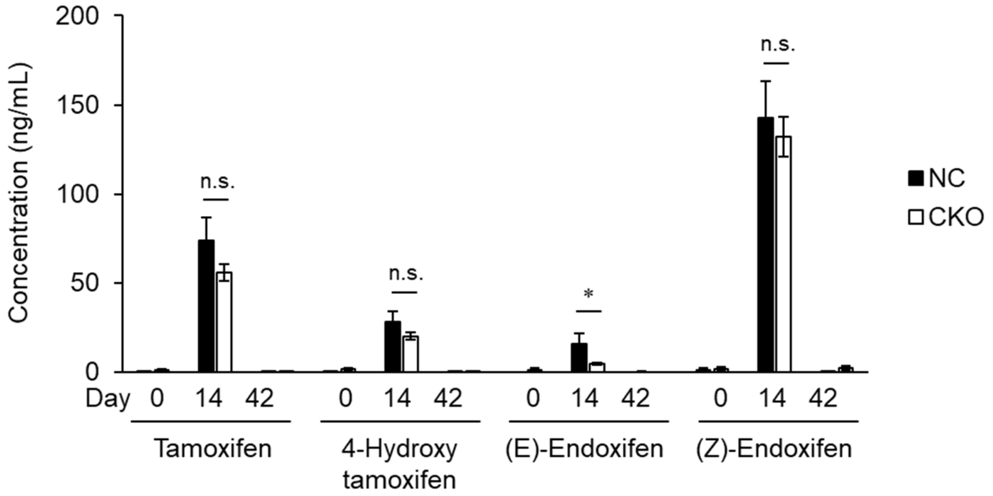

Figure S4. Quantification of tamoxifen and its metabolites in the serum. Tamoxifen and its metabolites, 4-hydroxytamoxifen and endoxifen, in mouse serum were quantified by LC-MS/MS as described in the Materials and Methods section. Data are presented as mean ± SEM ($n = 4$). Statistical significance of the differences in each metabolite was analyzed by two-way ANOVA and Tukey's test. *$p < 0.05$, n.s. not significant.

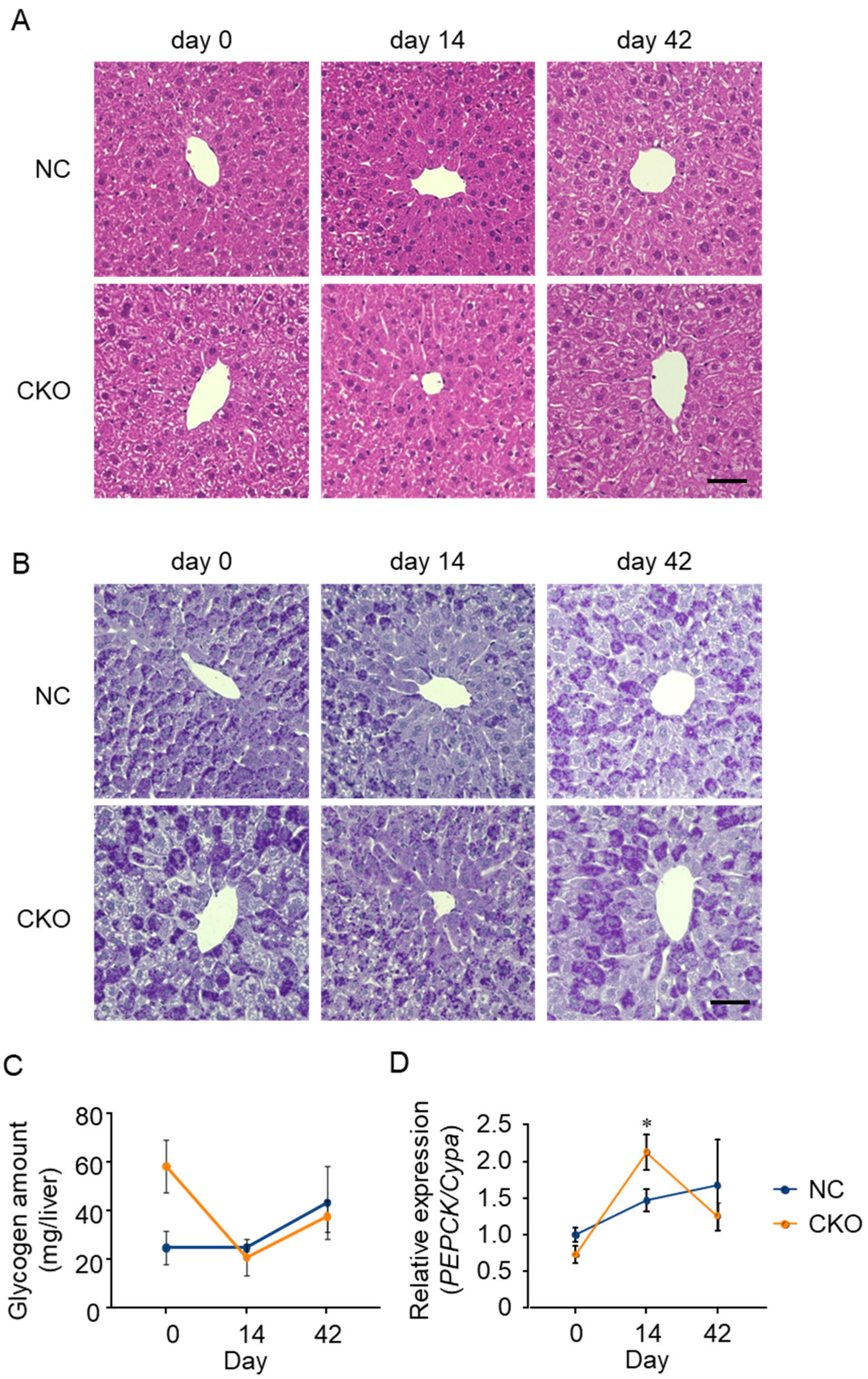

Figure S5. Histological analysis of *Gcn1* CKO mouse liver. (**A, B**) Representative images of H&E staining (**A**) and PAS staining (**B**) of CKO mice liver sections. Scale bar, 50 µm. (**C**) Quantification of liver glycogen content. The glycogen concentration in the liver was measured using a quantification kit. The total amount of glycogen per liver was expressed as mean ± SEM ($n = 3$ per group). Data were analyzed by two-way ANOVA and no significant difference was detected. (**D**) The expression of the gluconeogenic gene, phosphoenolpyruvate carboxykinase (*PEPCK*), was analyzed by RT-qPCR. Data are presented as mean ± SEM ($n = 3$ at days 0 and 42, $n = 6$ at day 14). Statistical analysis was carried out by two-way ANOVA and Tukey's test. *$p < 0.05$ compared with day 0.

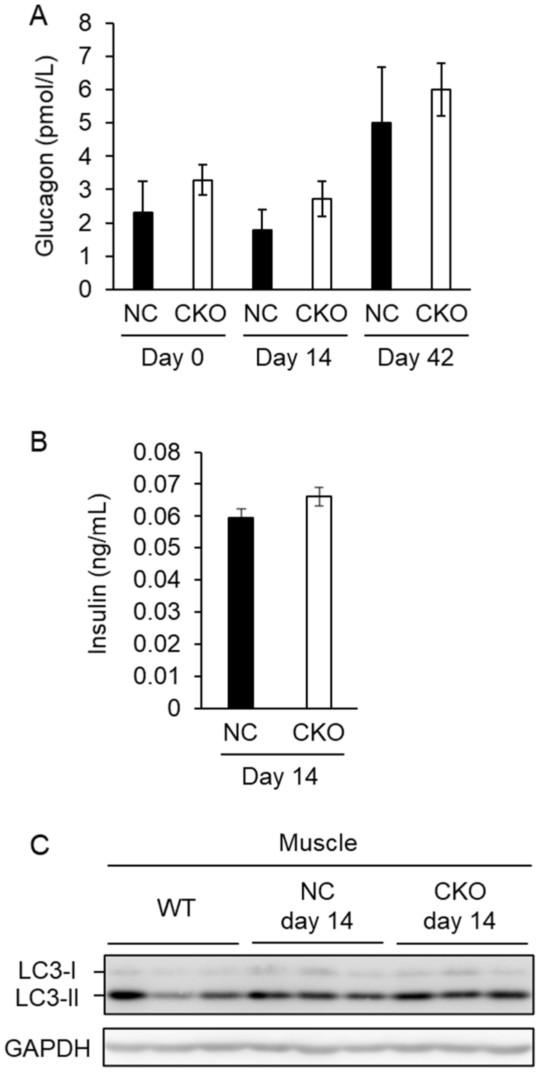

Figure S6. Absence of starvation in *Gcn1* CKO mice. (**A, B**) Quantification of glucagon (**A**) and insulin (**B**) in the serum. The concentration of the hormones was measured using a quantification kit. Data are expressed as mean ± SEM (n = 4 at day 14, n = 5 at day 0 and 42). Statistical analysis showed no significant difference. (**C**) Immunoblotting of LC3-I and its lipidation form (LC3-II) in muscle homogenates.

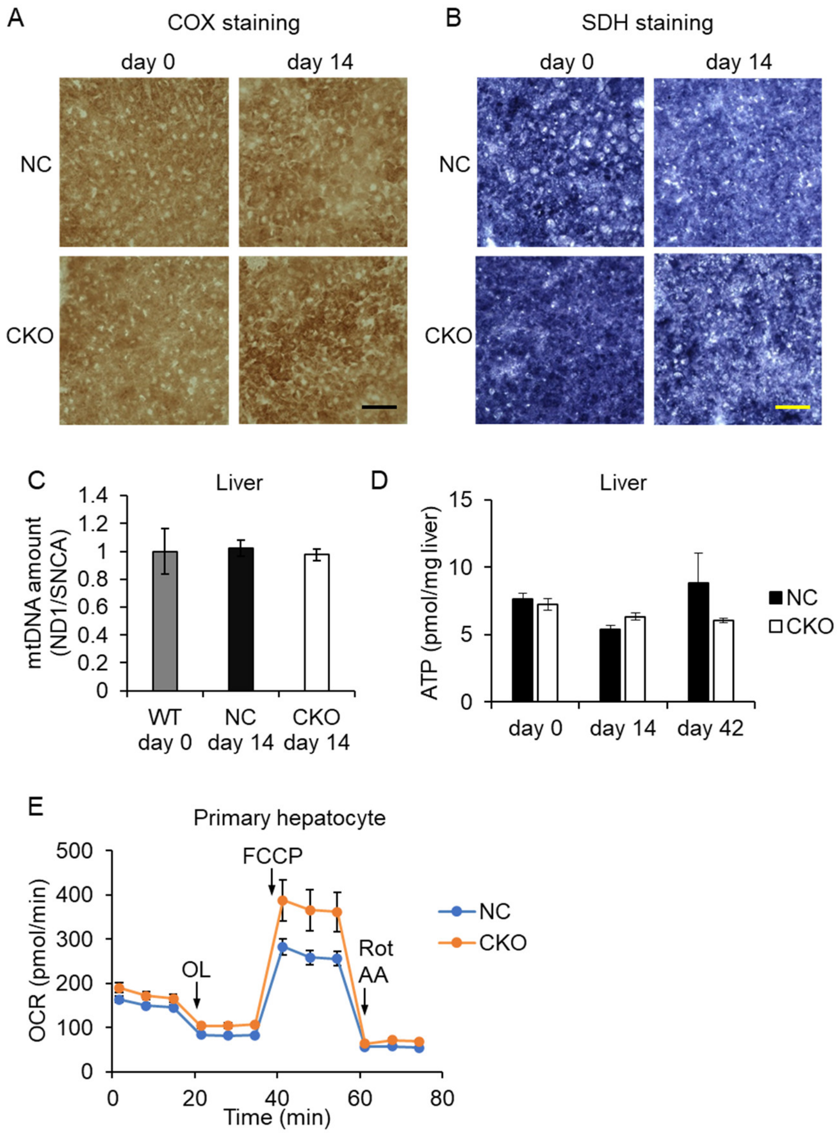

Figure S7. Analysis of mitochondrial functions. (**A, B**) Representative images of COX (**A**) and SDH (**B**) staining of frozen liver sections. Scale bar, 50 µm. (**C**) Quantification of mitochondrial DNA (mtDNA) copy number. mtDNA was quantified by qPCR of the mtDNA-encoded gene, *ND1*, which was normalized to the nuclear-encoded gene, *SNCA*. (D) Liver ATP measurement by a quantification kit. Data are expressed as mean ± SEM (n = 3 per group). Data were statistically analyzed by one-way ANOVA (**C**) and two-way ANOVA (**D**); no statistically significant differences were detected. (**E**) Oxygen consumption rate (OCR) measurement of CKO primary hepatocyte. Mitochondrial respiration was determined using Seahorse XF Cell Mito stress kit as described in Materials and Methods. Cells were treated with 1.5 µM oligomycin (OL), 1 µM FCCP, and 2 µM rotenone (Rot)/antimycin A (AA).

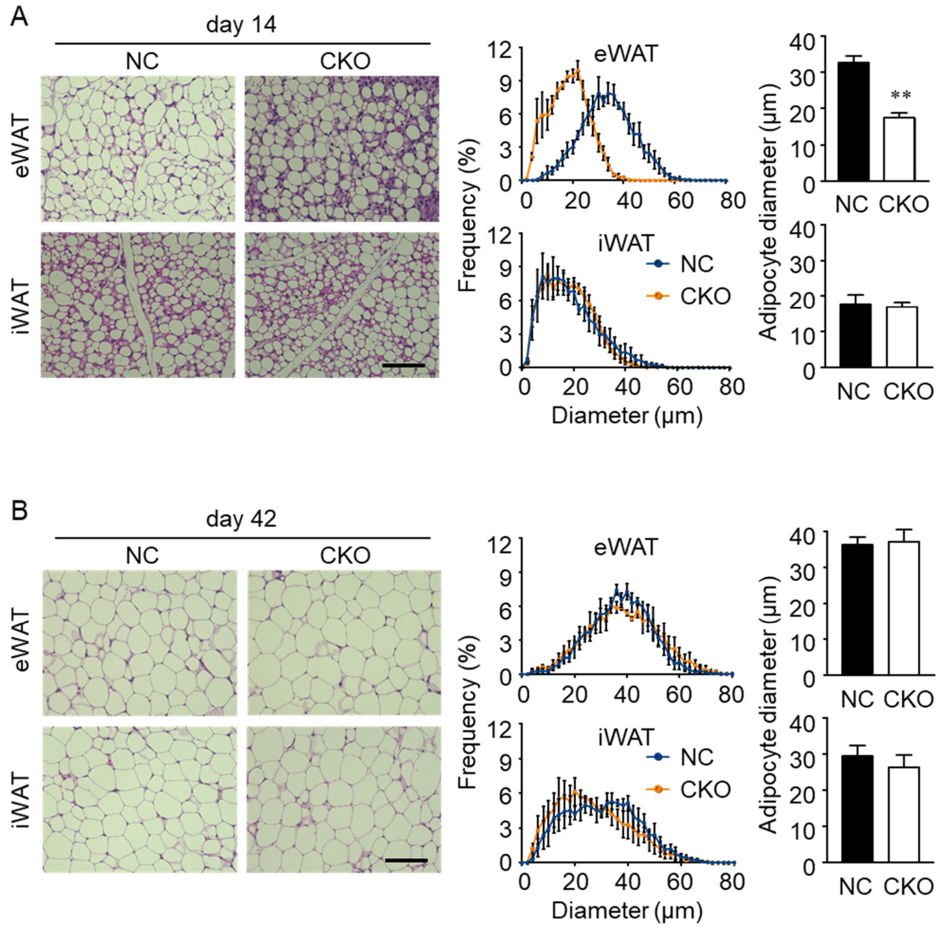

Figure S8. Change in adipocyte size in *Gcn1* CKO mice. (**A**, **B**) H&E staining of eWAT and iWAT tissue sections and cell size distribution in NC and CKO mice at day 14 (**A**) and day 42 (**B**). Adipocyte diameters were measured using ImageJ and the distribution frequency as well as mean ± SEM were shown (n = 3, ~2000 cells/sample). Data were statistically analyzed by t-test (**p < 0.01). Scale bar, 50 μm.

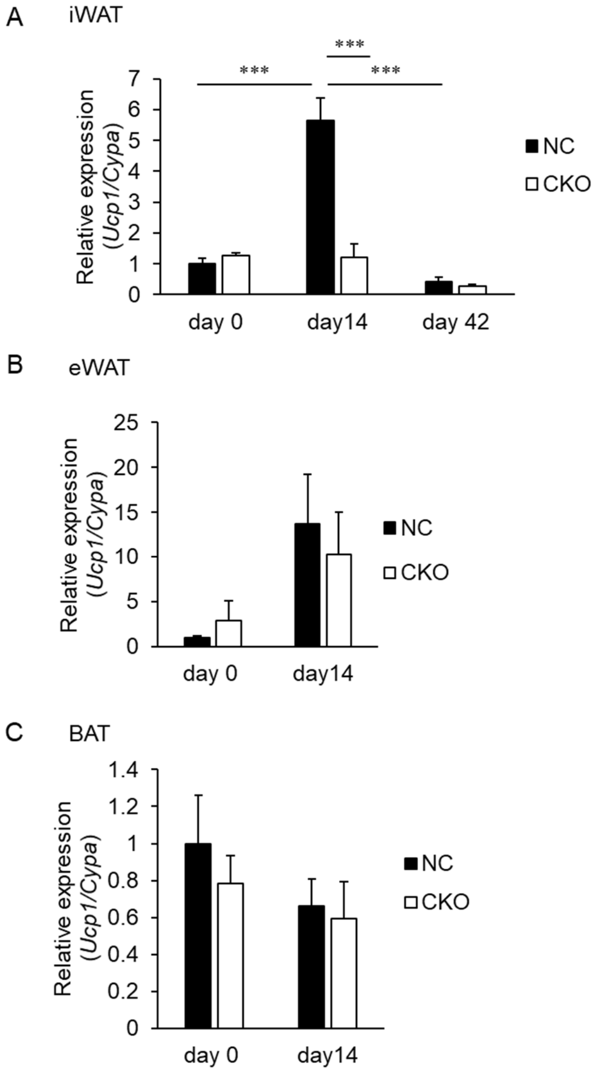

Figure S9. Ucp1 expression in adipose tissue after tamoxifen administration. (**A**-**C**) Ucp1 mRNA expression in inguinal white adipose tissue (iWAT) (**A**), epididymal white adipose tissues (eWAT) (**B**), and interscapular brown adipose tissues (BAT) (**C**). Ucp1 expression was analyzed by RT-qPCR as described in Materials and Methods. Data are expressed as mean ± SEM (n = 3 per group). Statistical analysis was carried out using two-way ANOVA and Tukey's test. ***$p < 0.001$.
